# Revealing the mechanisms and therapeutic potential of immune checkpoint proteins across diverse protein families

**DOI:** 10.3389/fimmu.2025.1499663

**Published:** 2025-04-28

**Authors:** Ran Liu, Xinyan Jiang, Ruijuan Dong, Yuting Zhang, Cong Gai, Peng Wei

**Affiliations:** School of Traditional Chinese Medicine, Beijing University of Chinese Medicine, Beijing, China

**Keywords:** immunotherapy, immune checkpoint proteins, tumor microenvironment specificity, co-suppressive pathways, protein families

## Abstract

Host immune responses to antigens are tightly regulated through the activation and inhibition of synergistic signaling networks that maintain homeostasis. Stimulatory checkpoint molecules initiate attacks on infected or tumor cells, while inhibitory molecules halt the immune response to prevent overreaction and self-injury. Multiple immune checkpoint proteins are grouped into families based on common structural domains or origins, yet the variability within and between these families remains largely unexplored. In this review, we discuss the current understanding of the mechanisms underlying the co-suppressive functions of CTLA-4, PD-1, and other prominent immune checkpoint pathways. Additionally, we examine the IgSF, PVR, TIM, SIRP, and TNF families, including key members such as TIGIT, LAG-3, VISTA, TIM-3, SIRPα, and OX40. We also highlight the unique dual role of VISTA and SIRPα in modulating immune responses under specific conditions, and explore potential immunotherapeutic pathways tailored to the distinct characteristics of different immune checkpoint proteins. These insights into the unique advantages of checkpoint proteins provide new directions for drug discovery, emphasizing that emerging immune checkpoint molecules could serve as targets for novel therapies in cancer, autoimmune diseases, infectious diseases, and transplant rejection.

## Introduction

Cancer is a leading cause of premature death worldwide, with its high mortality rate necessitating innovative therapeutic approaches (1). On April 4, 2024, A Cancer Journal for Clinicians published the most recent global cancer burden data for 2022, which revealed that lung cancer has overtaken breast cancer, once again becoming the most prevalent cancer worldwide (2).However, in many cases, durable remission is not achieved using treatments such as radiotherapy and chemotherapy. Therefore, the development of new therapies for the treatment of cancer is essential.

The tumor microenvironment is a highly heterogeneous ecosystem composed of tumor cells, immune cells, and other stromal cells. Immunotherapy is a promising emerging therapeutic modality for the treatment of many types of cancer (3). Recent advances in immunotherapy have demonstrated the potential of leveraging the immune system to combat cancer (4). Specifically, the immune biomarkers associated with checkpoint immunotherapy responses offer valuable insights into patients’ reactions to treatment (5). The immune microenvironment is a complex network comprising various immune cells, fibroblasts, cytokines, chemokines, and extracellular matrix proteins (6). These components interact extensively with each other and with tumor cells, thereby regulating cancer growth and progression. In certain cases, the immune system is capable of recognizing and attacking cancer cells, leading to tumor regression (7).

The idea that the immune system can recognize and control tumor growth dates back to 1893 when William Coley, a surgeon, used live bacteria as a form of immunotherapy to treat cancer. This early work laid the foundation for the modern understanding of cancer immunology (8). PD-1 was first discovered in 1991 by Yasuya Ishida in cDNA libraries of unstimulated and stimulated mouse T cells. It was subsequently named programmed cell death 1 (PD-1) due to its association with T cell apoptosis induced by specific stimuli.” (9).However, the effectiveness of cancer immunotherapy has been moderate due to its limited clinical efficacy. This limitation arises from the ability of tumor cells to evade recognition and elimination by the immune system, resulting in a tumor escape mechanism (10). Over the past few decades, significant progress has been made in understanding how cancer evades the immune system. This understanding has led to the development of new strategies aimed at blocking cancer’s immune escape, thereby enhancing the elimination of tumor cells (11). In some cases, the immune system fails to recognize and respond to cancer cells, allowing the tumor to evade detection and grow unchecked. Increasing evidence indicates that immune escape plays a crucial role in the survival and progression of tumors (12). Within the tumor microenvironment, tumor cells can recruit immunosuppressive cells, such as CD4+ T cells, which compromise the cytotoxic function of CD8+ T cells (13).

**Figure 1:**
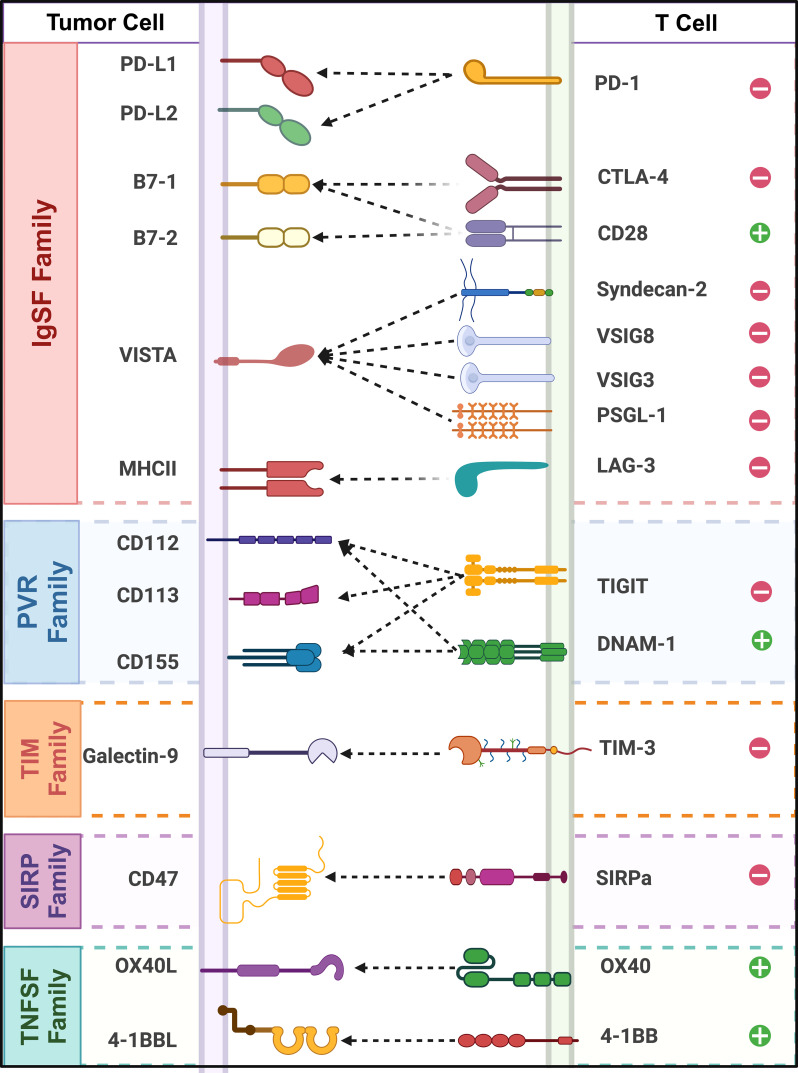
Classification and interactions of immune checkpoint receptors and ligands across different protein families. (This schematic illustrates key immune checkpoint molecules and their interactions between tumor cells and T cells. Tumor cell ligands (left) and their corresponding TCRs (right).”+” and “−” symbols represent stimulatory and inhibitory functions.).

Currently, extensive biological and medical research has categorized immune checkpoint proteins into distinct families based on their conserved domains, expanding the scope of immunotherapy research. (**Figure 1**) By reviewing the literature on immune checkpoints across various immune protein families, this paper aims to summarize the current research status of key immune checkpoints and offer new perspectives on cancer immunotherapy.

## IgSF family

The immunoglobulin superfamily (IgSF) is one of the largest and most versatile families of structural domains in animal genomes (14). IgSF protein genes account for more than 2% of human genes, making them the largest gene family in the human genome (15). Although the amino acid sequences of different family members vary considerably, the structural characteristics of the IgSF are traditionally defined by a few key site-specific residues critical for proper protein folding (16). During ontogeny, IgSF recognition molecules play essential roles in neuronal processes such as cell survival, migration, axonal guidance, and synaptic targeting (17). Many immune checkpoint proteins contain Ig structural domains or exhibit high homology with the V and C regions of immunoglobulins. PD-1, PD-L1, CTLA-4, BTLA, and VISTA are all part of the IgSF (18). All IgSF members contain 1-7 Ig-like structures, with each structure comprising approximately 70-110 amino acid residues (19). The secondary structure is a β-sheet formed by two anti-parallel β-strands, each composed of 3-5 amino acid residues, with 5-10 residues per strand. The hydrophobic amino acids within the β-sheet stabilize the folds (20). Techniques such as X-ray diffraction analysis and DNA sequence analysis have revealed that many cell membrane surface molecules and some protein molecules in the body share a similar peptide folding pattern with Ig structures (21). These molecules exhibit high homology with the variable (V) and conserved (C) regions of immunoglobulins, suggesting they may have evolved from a common ancestor (22). The genes encoding these polypeptide chains are referred to as the immunoglobulin gene superfamily, and their products are known as the IgSF (23).

We have summarized the structure and function of representative immune checkpoint proteins from different immune protein families, including the number of amino acids and Ig structural domains they contain (**Table 1**).

**Table 1:** Summary of receptor structures of representative immune checkpoints from different families.

Protein	Number of amino acids	Number of Ig domains	Function	
IgSF Family				
CD28	90	1	CD28 is a potent co-stimulatory receptor expressed on T cells, binding to its ligands CD80 and CD86. It plays a critical role in promoting T cell proliferation and enhancing the efficacy of T cell-mediated immune responses. The CD28 gene is located on chromosome 2q33.2.	(24)
PD-1	288	1	PD-1 interacts with its ligands, PD-L1 (B7-H1) and PD-L2 (B7-DC), in peripheral tissues, mediating immune suppression.	(25)
CTLA-4	223	1	Following TCR activation and CD28 co-stimulation, CTLA-4 translocates to the cell surface, where it competitively binds to CD80/CD86, outcompeting CD28. This interaction delivers inhibitory signals, suppressing T cell proliferation and activation.	(26)
VISTA	279	1	VISTA is both a T cell co-inhibitory ligand and a co-inhibitory receptor.	(27)
BTLA	336	1	Inhibition of T cell receptor (TCR) signaling pathway.	(28)
LAG-3	498	4	Induce the activation of Tregs and stimulate their immunosuppressive function.	(29)
PVR Family				
TIGIT	244	1	TIGIT can bind to CD155 of dendritic cells(DCs), triggering a cascade reaction indirectly hindering T cell function. It can also inhibit NK cell degranulation, produce cytokines, and mediate cytotoxicity against CD155+tumor cells.	(30)
CD96	482	3	CD96+NK cells exhibit a state of functional exhaustion, leading to IFN- γ And TNF- α Decreased secretion level.	(31)
CD155	417	1	CD155 serves as a ligand for the activating receptor DNAM-1, which is expressed on cytotoxic lymphocytes, including NK cells, and plays a key role in anti-tumor immune responses.	(32)
CD112	329	3	CD112R has a high affinity for CD112 on the surface of antigen-presenting cells(APCs) and some tumor cells, and when combined, it can inhibit the anti-tumor effects of T cells and NK cells.	(33)
CD112R	326	1		
CD226	336	4	Activation of cytotoxic T cells, NK cells, and platelet aggregation in mixed lymphocyte response of participants 1.	(34)
TIM Family				
TIM-1	346	1	TIM-1 can target and inhibit B cells, enhance anti-tumor CD8+ and CD4+T cell responses, and inhibit tumor growth, which is of great significance for cancer treatment.	(35)
TIM-3	281	1	TIM-3 and its ligands Gal-9, PtdSer, HMGB1, and CEACAM1. Binding leads to apoptosis of helper T cells (Th1/Th17), weakening activation and differentiation of other immune cells.	(36)
TIM-4	378	1	Tim-4 plays an important role in the proliferation of T helper cell 2 (Th2). Tim-4 binds to phosphatidylserine (PS) on the surface of apoptotic cells in a calcium dependent manner and mediates phagocytosis of apoptotic cells.	(37)
TNF Family				
TNF-α	157	0	TNF-α transmits information to the cell nucleus through specific receptors on the cell membrane, thus producing complex biological activities such as promoting cell proliferation and differentiation, immunomodulation, inflammation mediation and anti-tumor activity.	(38)
OX40	249	0	OX40 is a ligand-activated T-cell co-stimulator that mediates the survival and expansion of CD4+ and CD8+ T cells in a variety of animal models of autoimmunity, infectious disease, and cancer, and is also involved in the control of effector and memory T-cell responses.	(39)
4-1BB	255	0	Activation of 4-1BB co-stimulatory signaling by anti-4-1BB agonist or 4-1BBL transfection induces cell proliferation, cytokine expression, bactericidal activity, and support of T-cell effector function.	(40)
LIGHT	240	0	LIGHT is a member of the tumor necrosis factor (TNF) superfamily, a type II transmembrane glycoprotein that plays an important role in inflammatory diseases such as autoimmune hepatitis, urticaria, asthma, and nonalcoholic fatty liver.	(41)

Most IgSF members are membrane proteins located on the surface of lymphocytes, playing a crucial role in various immune activities (42). The discovery of the Ig structure in invertebrate cellular adhesion molecules, which lack an immune system, suggests that Ig proteins originally functioned as adhesion molecules during early evolution, and later adapted to serve immune functions (43). The identification of Ig proteins as intermediaries in the evolution of cellular slime molds in invertebrates, followed by the discovery of their immune functions in vertebrates, indicates that the multifunctional nature of IgSF was likely created through gene duplication and subsequent divergence. Japanese scientist Susumu Tonegawa was awarded the Nobel Prize in Physiology or Medicine in 1987 for his groundbreaking research on the structure of immunoglobulin genes (44).

### PD-1

Programmed death-1 (PD-1) is a crucial immunoregulatory receptor expressed by activated T cells. PD-1 is a type I transmembrane protein composed of 288 amino acids and is a member of the CD28/CTLA-4 family of T cell regulators. The protein structure includes an extracellular IgV domain, a transmembrane domain, and an intracellular tail (45). The intracellular tail contains two phosphorylation sites within the immune receptor tyrosine-based inhibitory motif (ITIM) and the immune receptor tyrosine-based switch motif (ITSM), indicating that PD-1 negatively regulates TCR signaling (46). PD-1 primarily binds to its ligands, PD-L1 (B7-H1) and PD-L2 (B7-DC), to mediate immunosuppression. PD-L1 and PD-L2 are expressed by tumor cells, stromal cells, or both (25). The discovery and application of PD-1 indicates that the research of tumor therapy has entered a new stage (47).

**Figure 2:**
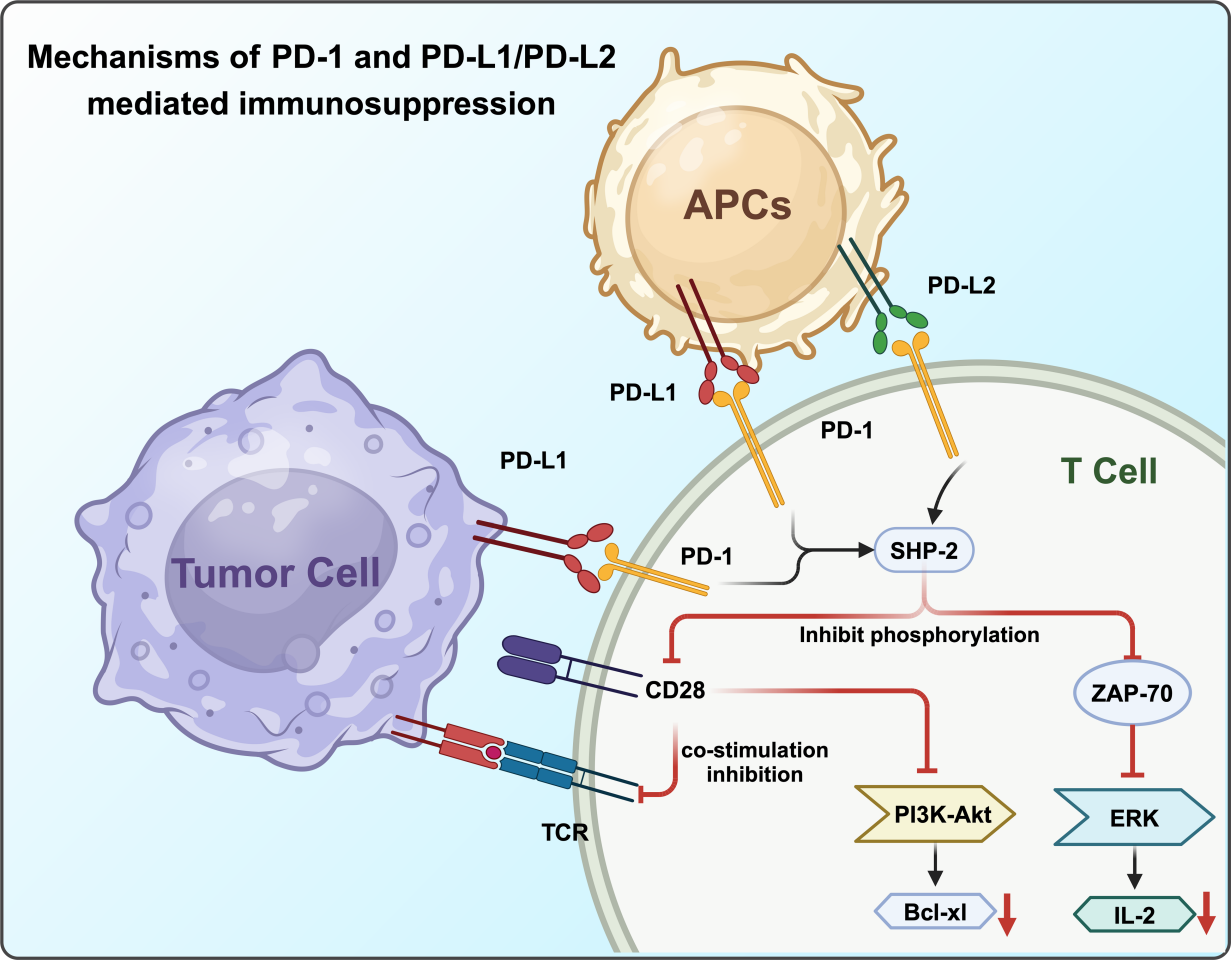
Mechanisms of PD-1 and PD-L1/PD-L2 mediated immunosuppression.

In the presence of PD-L1, PD-1 and CD28 colocalize at the center of TCR-enriched regions. PD-1, upon activation, recruits the protein tyrosine phosphatase SHP-2, which reduces CD28 phosphorylation and suppresses TCR signaling intensity (48). The PD-1-mediated dephosphorylation of CD28 significantly disrupts PI3K recruitment to the TCR signalosome, leading to decreased activation of the PI3K/AKT pathway and reduced expression of its transcriptional targets, such as Bcl-xL. Furthermore, SHP-2 not only blocks CD28 co-stimulatory signaling but also inhibits TCR-mediated phosphorylation of ZAP70, impairing ERK activation and subsequent IL-2 production and amplification (25) (**Figure 2**).

PD-L1 is expressed by APCs, including human peripheral blood interferon-stimulated monocytes that activate human and mouse DCs. It is also expressed in non-lymphoid tissues such as heart and lung (49). Monoclonal antibodies can restore the anti-tumor activity of CD8+ T cells by blocking the inhibitory signaling pathways (50). However, targeting a single immunosuppressive pathway may not completely eliminate tumors. Another ligand of PD-1, PD-L2, acts as a T cell inhibitory receptor (51).

Although much research has focused on the PD-1/PD-L1 interaction, PD-L2 (B7-DC), a member of the B7 family, was identified in DCs in 2001 (52). Binding of PD-L2 to PD-1 significantly inhibited TCR-mediated CD4+ T cell proliferation and cytokine production, leading to the discovery of the overlapping functions of PD-L1 and PD-L2 (53).While initially thought to be expressed primarily in macrophages in the presence of interleukins, recent studies have shown that PD-L2 is expressed in various tumor cells depending on the tumor microenvironment (54). The activation of the PD-1 signaling pathway can lead to T cell apoptosis and exhaustion, resulting in immunosuppression due to T cell dysfunction. Immune checkpoint blockade against PD-1 inhibits its interaction with both PD-L1 and PD-L2 (55).

Compared with PD-L1, the expression of PD-L2 is relatively limited, mainly found on APCs such as activated macrophages and DC (56). Although the interaction affinity between PD-L2 and PD-1 is several times higher than that of PD-L1, PD-L2 is usually expressed at lower levels, making PD-L1 the primary ligand. Consequently, the PD-1/PD-L1 signaling pathway remains a major focus of research (57).

To date, five anti-PD-1/PD-L1 drugs have received approval from the US Food and Drug Administration (FDA). These include anti-PD-1 drugs such as pembrolizumab (Keytruda; Merck & Co., Inc., Kenilworth, NJ, USA) and nivolumab (Opdivo; Bristol-Myers Squibb Company, New York, NY, USA), as well as anti-PD-L1 drugs like atezolizumab (Tecentriq; Genentech, Inc., South San Francisco, CA, USA), avelumab (Bavencio; EMD Serono, Inc., Merck KGaA, Darmstadt, Germany), and durvalumab (Imfinzi; AstraZeneca UK Limited, Cambridge, UK).Of these, pembrolizumab and nivolumab have been used with good efficacy in a variety of diseases (**Table 2**).

**Table 2:** Summary of selected anti-PD-1 and anti-PD-L1 drugs approved for marketing by the FDA.

Drug	Diseases	Pathways of drug action	Reference
PD-1			
Pembrolizumab	Colorectal cancer	Pembrolizumab binds to and blocks PD-1 on lymphocytes, thereby modulating their ability to target and attack colorectal cancer cells.	(58)
	Melanoma	Anti-PD-1 antibodies exert their effects by binding to PD-1 receptors on T cells, as well as on B cells and NKs, including those in melanoma.	(59)
	NSCLC	Pembrolizumab enhances the immune system’s ability to recognize non-small cell lung cancer (NSCLC) tumor cells in immunotherapy, leading to an anti-tumor response and inducing apoptosis.	(60)
	Hodgkin lymphoma	Pembrolizumab prevents Hodgkin’s lymphoma cells from evading immune destruction by blocking the interaction between the T cell regulatory protein programmed cell death-1 (PD-1) and its ligands, programmed cell death ligand 1 (PD-L1) and programmed cell death ligand 2 (PD-L2).	(61)
Nivolumab	Melanoma	Nivolumab is a high-affinity, fully human immunoglobulin G4 (IgG4) antibody that specifically targets programmed cell death ligand 1 (PD-L1), inhibiting its binding to PD-1 and CD80.	(62)
	NSCLC	PD-L1 is expressed in approximately 50% of NSCLCs (NSCLC), primarily in advanced squamous subtypes. Nivolumab binds to the PD-1 receptor and blocks its interaction with PD-L1 and PD-L2, thereby releasing the inhibition of immune responses mediated by the PD-1 pathway.	(63)
	Urothelial cancer	Nivolumab exhibits a high affinity for PD-1 and can competitively inhibit the binding of the PD-L1 receptor to PD-1 in urothelial cancer cells.	(64)
PD-L1			
Atezolizumab	NSCLC	atezolizumab restores the T cells’ ability to detect and attack cancer cells. This mechanism is particularly effective in tumors with higher PD-L1 expression, enhancing immune surveillance and leading to tumor cell destruction.	(65)
	Triple-Negative Breast Cancer(TNBC)	Since TNBC lacks hormone receptors and HER2 expression, it doesn’t respond to hormonal or HER2-targeted therapies, making immune checkpoint inhibitors(ICIs) like atezolizumab more effective options.	(66)
	Urothelial carcinoma	Atezolizumab treats urothelial carcinoma by targeting PD-L1, a protein on the surface of tumor cells. PD-L1 binds to PD-1 receptors on T cells, preventing the immune system from attacking cancer cells.	(67)
Durvalumab	NSCLC	Durvalumab is an anti-PD-L1 monoclonal antibody that blocks this interaction, reactivating T cells so they can recognize and attack the cancer cells. This approach boosts the body’s immune response against the tumor and is particularly effective in patients with stage III NSCLC who have not shown disease progression after chemotherapy and radiation.	(68)
	Urothelial Carcinoma	Clinical studies have shown that durvalumab is particularly effective for patients who are cisplatin-ineligible or who have progressed after platinum-based chemotherapy. In such cases, durvalumab has demonstrated benefits in overall response rates and survival, making it a critical alternative for patients with aggressive urothelial cancer.	(69)
Avelumab	Merkel Cell Carcinoma (MCC)	Avelumab treats Merkel Cell Carcinoma (MCC) through immune checkpoint inhibition, specifically by targeting and blocking the PD-L1 protein on tumor cells. Normally, PD-L1 interacts with the PD-1 receptor on T cells, leading to immune suppression that allows cancer cells to evade immune detection.	(70)
	Renal Cell Carcinoma (RCC)	Avelumab is used in combination with axitinib (a tyrosine kinase inhibitor) as a first-line treatment for advanced renal cell carcinoma, enhancing immune activity against the tumor.	(71)

There is growing evidence that drugs targeting immune checkpoints can provide significant clinical benefits, including prolonged response and survival. Monoclonal antibodies targeting the programmed death-1/programmed death ligand-1 (PD-1/PD-L1) immune checkpoint pathway—such as Nivolumab, Pembrolizumab, Atezolizumab, Avelumab, and Durvalumab—have demonstrated considerable efficacy and offer new therapeutic opportunities for many cancer patients. However, reports indicate that the effectiveness of these monoclonal antibodies is often limited due to the emergence of intrinsic or acquired resistance mechanisms and a lack of durable responses in some patients with melanoma (72).

### CTLA-4

CTLA-4, or cytotoxic T lymphocyte-associated antigen-4 (also known as CD152), is located on band 33 (2q33) of the long arm of chromosome 2 (73). It exhibits high homology with the costimulatory receptor CD28 found on T cells (74). CTLA-4 is a membrane protein with a relatively short intracellular domain consisting of only 36 amino acids. This domain contains an immune tyrosine inhibitory motif (ITIM), which contrasts with the immune tyrosine activating motif (ITAM) present in CD28 (75).

Both CTLA-4 and CD28 are expressed on the surface of activated CD4+ and CD8+ T cells and are members of the IgSF. They share the same ligands, CD86 (B7-2) and CD80 (B7-1). The binding of CD28 to B7-1/2 generates stimulatory signals that promote cytokine IL-2 mRNA production, cell cycle entry, T cell activation, helper T cell differentiation, and immunoglobulin isotype switching (76). In contrast, CTLA-4 inhibits T cell activation by competitively binding to B7-1 and B7-2, which are normally bound by CD28. This competitive binding downregulates the TCR signaling pathway, reduces IL-2 secretion, and serves as a negative regulator of T cell responses (77).

Regulatory Tregs further inhibit T cell activation by down-regulating CD80/CD86 expression via CTLA-4, thereby disrupting the CD28 signaling pathway. CTLA-4 inhibitors exert anti-tumor effects by preventing Tregs from down-regulating CD80/86 expression and depleting Tregs through antibody-dependent cell-mediated cytotoxicity (ADCC) and phagocytosis (ADCP) (78). This increases the infiltration of CD4+/CD8+ T cells into tumor tissues and enhances the clonality of memory T cells (79).

Compared with PD-1/PD-L1 monoclonal antibodies, CTLA-4 monoclonal antibody drugs, despite being introduced and clinically applied earlier, are relatively limited in variety and are primarily approved for use in combination with other monoclonal antibodies. Currently, the only CTLA-4 inhibitors approved by the US FDA are ipilimumab and tremelimumab. Of these, only ipilimumab is approved by the FDA for the treatment of melanoma, kidney cancer and advanced metastatic colorectal cancer (80–82).

The goal of cancer immunotherapy should remain the complete and safe eradication of cancer from the patient’s body (83). Achieving this goal requires a unique immunotherapy regimen based on the biology present in a given patient’s body, and some patients may require only a single therapy, while others may require a combination of therapies (84). The introduction of CTLA-4 inhibitors has deepened the understanding of immunotherapy among clinicians and increased interest in dual immunotherapy (85). A Phase II clinical trial (CheckMate-069) demonstrated that the combination therapy of nivolumab (a PD-1 monoclonal antibody) and ipilimumab (a CTLA-4 monoclonal antibody), also known as “O+Y,” resulted in a higher objective response rate (ORR) and complete response rate in BRAF wild-type patients compared to ipilimumab monotherapy (61% vs. 11% and 22% vs. 0%, respectively) (86). Additionally, in BRAF-mutant patients, combination therapy significantly prolonged median progression-free survival (mPFS) (8.5 months vs. 2.7 months). Another combination therapy, “D+T” (Durvalumab, a PD-L1 monoclonal antibody, and Tremelimumab, a CTLA-4 monoclonal antibody), has been applied in the first-line treatment of advanced hepatocellular carcinoma. We have summarized the approved combination therapies and their effects across different diseases (87) (**Table 3**).

**Table 3:** Summary of selected anti-PD-1 combined with anti-CTLA-4 drugs approved for marketing by the FDA.

Diseases	Pathways of action of co-immune drugs	FDA approved time	References
Effect of combination therapy with “O+Y”(Nivolumab and Ipilimumab)			
Melanoma	In BRAF wild-type patients, combination therapy increased the ORR and complete response rate by 50%, while in BRAF mutant patients, the mPFS was significantly extended by 5.8 months with the “O+Y” combination therapy.	2016	(88)
Non-small cell lung cancer	The latest data from the CheckMate 227 study reaffirmed the significant survival benefit of “O+Y” for the first-line treatment of metastatic NSCLC. The 6-year OS rates of the combination therapy were superior to those of the chemotherapy group (9% increase in 6-year OS rates for patients with PD-L1 ≥1%; 11% increase in 6-year OS rates for patients with PD-L1 <1%). Median OS was prolonged by 4.8 months in the nivolumab combined with ipilimumab group.	2018	(89)
Malignant pleural mesothelioma	The “O+Y” combination therapy significantly extends the median overall survival (mOS) in patients with malignant pleural mesothelioma (MPM), with a 14% increase in the 2-year mOS. After a follow-up period of 35.5 months, which is 1 year after discontinuation of treatment, the 3-year mOS rate is 1.5 times higher than that achieved with chemotherapy alone, and the risk of death is reduced by 27%.	2020	(90)
Renal cell carcinoma	The combination therapy of “O+Y” extended the OS of patients with medium to high-risk renal cell carcinoma from 26.6 months to 47 months, reducing the risk of death by 34%. It also prolonged PFS from 8.3 months to 12 months, reducing the risk of progression or death by 24%.	2021	(91)
Effect of combination therapy with “D+T”(Durvalumab and Tremelimumab)			
Hepatocellular carcinoma	The combination of “D+T” prolongs mOS by 2.6 months, mPFS by 3.8 months and 4.1 months, ORR by 15%, and overall 3-year survival rate by 10.5% in the treatment of hepatocellular carcinoma.	2022	(92)

CTLA-4 inhibitors, such as ipilimumab, have been available for several years but have not achieved significant breakthroughs in monotherapy for various solid tumors. This may be due to an incomplete understanding of CTLA-4’s mechanism of action and its relationship with the PD-1/PD-L1 signaling pathway. Additional factors, including variations in IgG antibody types, pH-dependent antibodies, and antigenic epitopes, complicate achieving the expected clinical efficacy of these drugs. However, the development of PD-1/CTLA-4 combination therapies may address these challenges.

Given the complexity, uncertainty, and associated risks of immunotherapy, along with the notable variability in immune checkpoint therapy effectiveness among patients with different clinical profiles, there is a need for more comprehensive evidence-based medicine. Precise biomarkers are required to identify patient populations that are most likely to benefit from immunotherapy, thereby mitigating risks. The use of CTLA-4 inhibitors across various tumor types and treatment stages should be guided by evidence-based medicine and relevant clinical guidelines.

### VISTA

VISTA, also known as V-type immunoglobulin domain-containing suppressor of T cell activation or PD-1H, is an immune checkpoint protein that plays a critical role in suppressing T cell-mediated anti-cancer responses (93). The VISTA protein spans 279 amino acids, including a 162-aa extracellular domain, a 21-aa transmembrane domain, and a 96-aa cytoplasmic domain (94). The cytoplasmic domain contains multiple phosphorylation sites for casein kinase 2 and protein kinase C. Similar to PD-1 and CTLA-4, VISTA inhibitors have the potential to enhance the immune system’s ability to eliminate tumors. The immunoglobulin variable (IgV)-like folding in VISTA’s extracellular domain includes two additional disulfide bonds and an extended loop with additional helices, forming a clinically relevant continuous binding epitope for antiviral antibodies. This antibody-binding region is closely related to the Ig domain (VSIG3), a significant ligand for VISTA (95).

Compared to peripheral lymph nodes, VISTA is more abundantly expressed in MDSCs within the tumor microenvironment (TME). Under the hypoxic conditions of the TME, VISTA expression is significantly upregulated, leading to the suppression of TLR signaling and inhibition of cell migration (96). By reprogramming myeloid cells, VISTA reduces the production of pro-inflammatory cytokines such as TNF-α while increasing anti-inflammatory mediators like IL-10, thereby enhancing the immunosuppressive function of myeloid cells. Additionally, VISTA promotes peripheral immune tolerance by facilitating activation-induced T cell death (27).

VISTA may also be crucial in regulating inflammation and autoimmune diseases, such as graft-versus-host disease (GVHD), acute hepatitis, encephalitis, and lupus (27). Additionally, VISTA acts as a co-inhibitory receptor on T cells, significantly modulating antigen-specific CD4+ T cell responses and protecting mice from GVHD, acute hepatitis, and asthma (97).VISTA is primarily expressed on CD45+ cells located near tumors and is also present in the hematopoietic system, with notable expression in myeloid cell compartments (98). It is most abundantly expressed on myeloid cells and DCs, and less so on T cells. The extracellular domain of VISTA contains numerous histidine residues, which confer pH-dependent functionality. Specifically, histidine residues interact with ligands when the extracellular pH decreases from 7.4 to 6.0, a condition found in the tumor microenvironment, lymph node regions, or healing wounds (99). Five ligands of VISTA - PSGL-1, Syndecan-2, LRIG-1, VSIG8, and VSIG3 - were found to bind differently at pH values of 6.0 and 7.4 (100).

**Figure 3:**
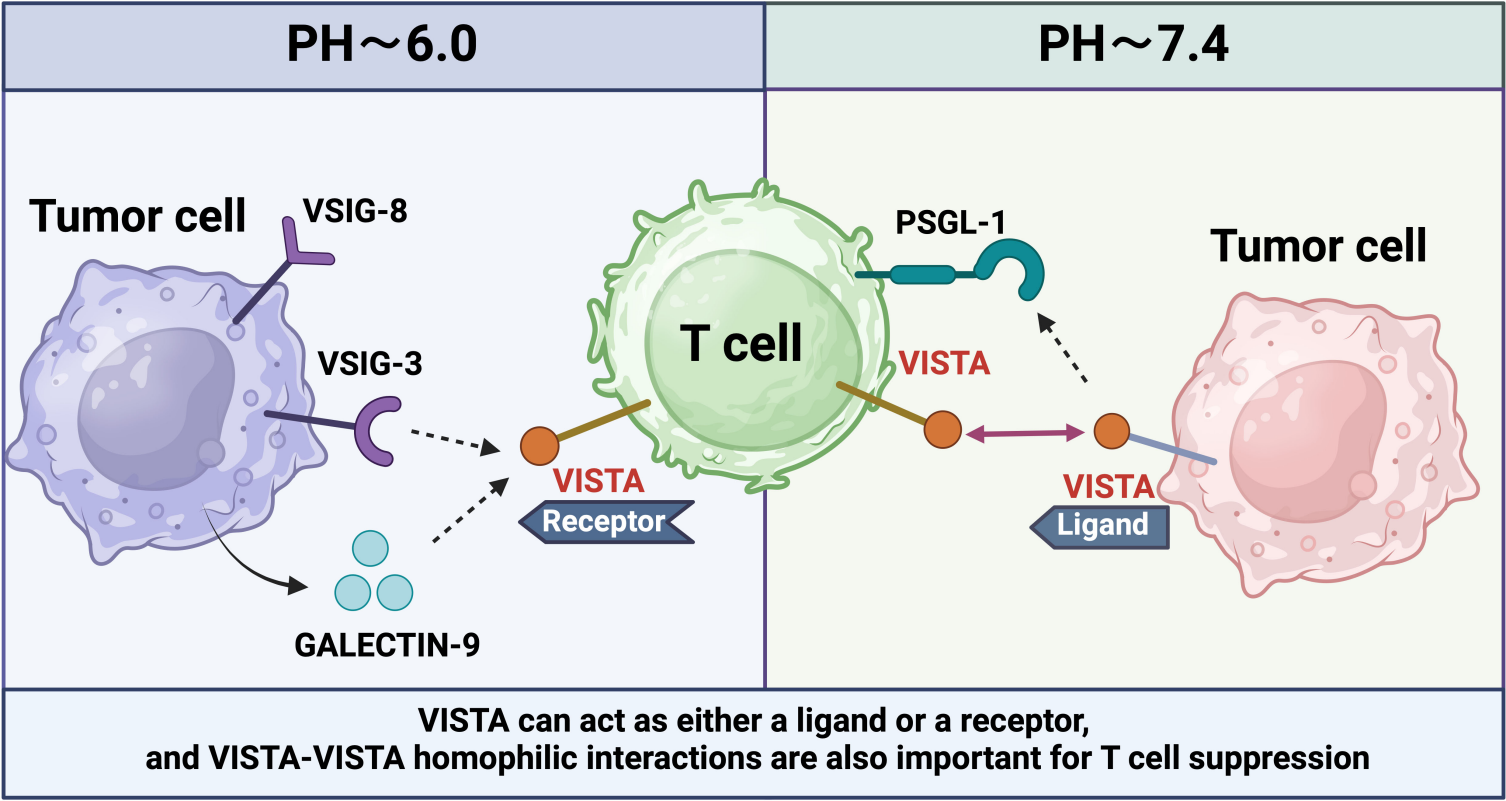
VISTA is expressed on T cells or tumor cells in different PH environments to regulate different immune responses.

Antibodies that selectively bind to and block interactions in acidic environments can potentially reverse VISTA-mediated immunosuppression *in vivo*. PSGL-1, expressed on T and B cells, myeloid cells, and DCs, can inhibit T cell proliferation and promote a depletion phenotype, although the precise mechanism by which VISTA mediates this effect remains unclear (101). This selective interaction and inhibition of T cells at acidic pH values are mediated by histidine residues along the periphery of the VISTA extracellular domain, which facilitate binding to the adhesion and co-inhibitory receptor PSGL-1 (102). To illustrate the structural and functional diversity of immune checkpoint molecules, we have depicted the complex mechanisms by which these molecules regulate immune responses, particularly in the context of cancer and autoimmune diseases (**Figure 3**).

Additionally, the interactions between VISTA and its ligands VSIG3 and VSIG8 inhibit T cell activation and effector functions. VISTA also induces the formation of regulatory Tregs from human CD4+ T cells (103). Furthermore, VISTA promotes the inhibition of myeloid cells and tolerogenic DCs by interfering with the MAPK and NF-kB pathways within the TLR signaling cascade (104). Early studies utilizing rat anti-mouse viral antibodies in combination with anti-PD-1 or anti-PD-L1 antibodies have demonstrated efficacy across various mouse tumor models (105). In these models, selective blockade of the interaction with PSGL-1 at pH 6.0, rather than at pH 7.4, offers additional therapeutic benefits against PD-1. These pH-selective antibodies accumulate in the acidic tumor microenvironment rather than in major viral expression sites like the spleen. Compared to non-pH-selective antibodies, pH-selective antibodies have shown improved safety and efficacy in non-human primates (106). The development of pH-selective VISTA antibodies represents a promising new strategy for cancer therapy.

The dual role of VISTA as both a receptor and a ligand has been demonstrated through its ability to engage in homologous interactions. Homologous VISTA-VISTA binding facilitates the phagocytosis of apoptotic cells by macrophages, thereby contributing to the clearance of apoptotic cells from the internal environment. A prior study utilizing VISTA-Ig fusion protein to treat wild-type (WT) T cells and VISTA knockout (KO) T cells *in vitro* revealed that VISTA KO T cell proliferation was less affected by the VISTA-Ig protein compared to WT T cells (107).

### B7-H3

B7 homologous protein 3 (B7-H3, also known as CD276), a newly discovered member of the B7 family, is an immunomodulatory protein with co-stimulatory/co-inhibitory effects and is an attractive and promising target for cancer immunotherapy, playing a dual role in the immune system (108).

B7-H3 is a type I transmembrane protein containing 316 amino acids with a molecular weight of ~45-66 kDa, which was first discovered in 2001 from a cDNA library derived from human DCs (109).The human B7-H3 gene is located on chromosome 15 and the mouse B7-H3 gene is localized on chromosome 9 (110).Upper B7-H3 shares 20-27% amino acid homology with other B7 family members (111).B7-H3 is abundantly expressed on the surface of tumor cells, with limited expression in normal cells, and is also involved in the formation of the tumor microenvironment (TME) (112).

TREM-like transcript 2 (TLT-2) was identified as a potential receptor for B7-H3 (113). However, TLT-2 may not be the only receptor for B7-H3. In contrast to other immune checkpoints, B7-H3 also regulates cancer cell invasiveness through various non-immune pathways (114).A study in 2019, using a new interactome platform with high-throughput data, identified interleukin-20 receptor subunit alpha (IL20RA) as the first target for B7-H3 binding (115).The significance of IL20RA as a cancer biomarker has been investigated and overexpression of IL20RA promotes cancer stemness through the transcription factor SOX2 and suppresses immunity through increased PD-L1 expression (116).In addition, a 2021 study detected phospholipase A2 receptor 1 (PLA2R1) as another high-level binding protein among all single-channel transmembrane proteins and their exogenous sources based on the leaflet vesicle interactions group platform (117).

B7-H3 was initially found to be an immune co-stimulant (118), in which B7-H3-Ig induced the proliferation of CD4+ and CD8+ T cells and increased the secretion of interferon γ thereby enhancing T cell activity.B7-H3 also enhances T cell activity by promoting the production of IL-10, TGF-β1.In addition, the positive correlation between the expression of FOXP3+ tregs and B7-H3 favoring the immune system to suppress the tumor microenvironment (119, 120). On the other hand, B7-H3 inhibited the secretion of IFN-γ, IL-2, perforin, and granzyme B, thereby suppressing the activity of CD4+ T cells, CD8+ T cells, γδ T cells, CAR-T cells, Vδ2 T cells, T17 cells, CD3+ T cells, NK cells, macrophages, neutrophils, and DCs (121–124), while B7-H3 regulated the differentiation of tumor-associated macrophages, promotes polarization of type 2 macrophages, and converts the M1 phenotype to the M2 phenotype (125). B7-H3 triggers different signaling cascades to activate downstream molecules that contribute to the malignant behavior of cancer cells, e.g., B7-H3 activates signaling pathways such as ERK, PI3K, and Stat3 in cancer cells, leading to accelerated cell proliferation and tumor growth (126).

Studies have shown that B7-H3 is abundantly expressed in mouse and human adipose tissue and preferentially expressed in adipocyte progenitor cells (APs), and knockdown of the gene leads to spontaneous obesity in mice, demonstrating a role for B7-H3 in adipocyte progenitor cell differentiation, lipid oxidation, and obesity, in addition to its immunomodulatory function (127). In addition, this study revealed a plausible link between diabetes mellitus (DM) and B7-H3. B7-H3 knockout mice exhibited an increased propensity for obesity and related metabolic syndrome. In another study, patients with type 1 diabetes had significantly higher serum B7-H3 levels than healthy controls. Given this evidence, the role of B7-H3 in the pathologic process of diabetes needs to be further explored (128).

However, the multifaceted role of B7-H3 in the tumor microenvironment has been extensively studied, and B7-H3 has been found to induce malignant behaviors and promote tumor progression through complex pathways. Role of B7-H3 in Tumor Cells, T Cells, DCs, NK Cells, CAFs, Neutrophils, and Endothelial CellsB7-H3 is a key regulator of the tumor microenvironment, and a valuable immunotherapeutic target (129).

### LAG-3

Lymphocyte activation gene 3 (LAG-3) is a cell surface inhibitory receptor that regulates T cell activation and effector functions (130). LAG-3, a member of the IgSF, is encoded on human chromosome 12, recognized as a third-generation inhibitory receptor, it is considered a promising therapeutic target following PD-1 and CTLA-4. First identified by Triebel et al. in 1990 on activated human NK and T cells, LAG-3 has gained attention as an immune checkpoint molecule and a key target in cancer immunotherapy (131).

LAG-3 is a type I transmembrane protein weighs approximately 70 kDa and comprises 498 amino acids, spanning extracellular, transmembrane, and cytoplasmic regions. Its expression correlates with tumor prognosis and is found on effector T cells and regulatory Tregs, influencing T lymphocyte and APC signaling (132). The LAG-3 gene is located near the CD4 gene and shares structural similarities, suggesting both evolved from a common ancestral IgSF-encoding gene (133).

The cytoplasmic tail of LAG-3 is crucial for its negative signal transduction function within the cell, its loss completely abolishes this function. The cytoplasmic region of LAG-3 contains three conserved motifs. The first region includes serine phosphorylation sites, the second contains a single lysine residue within the unique “KIEELE” motif, and the third includes glutamate-proline (EP) repeat sequences (134). The absence of the KIEELE motif completely disrupts LAG-3 function on CD4 T cells, underscoring its critical role in inhibiting signal transduction (135).

LAG-3 is expressed in NK cells, B cells, and plasmacytoid DCs. Its expression is induced by TCR activation or cytokines such as IL-12, IL-27, IL-15, IL-2, and IL-7 (136). LAG-3 may serve as a depletion marker similar to PD-1 in CD8+ T cells, particularly in response to repeated antigen stimulation during chronic viral infections or cancer (137). Evidence suggests that LAG-3 interferes with common pathways involved in CD4 and CD8 activation and regulates the activation and expansion of memory T cells (138).

LAG-3 is associated with the TCR: CD3 complex on the T cell membrane, where it negatively regulates TCR signaling, leading to the suppression of cell proliferation and cytokine secretion (139). The co-participation of LAG-3 and CD3 in the immune synapse is essential for attenuating TCR signaling (140). Additionally, the simultaneous engagement of LAG-3/TCR with their respective ligands inhibits TCR: CD3-dependent intracellular calcium flux, further dampening TCR-dependent signaling cascades and suppressing T cell responses (131).

MHC class II (MHC-II) molecules are recognized as typical ligands for LAG-3. These molecules, which are abnormally expressed by APCs or melanoma cells, stably interact with LAG-3 through its D1 domain, exhibiting significantly higher affinity than with CD4 (141). This interaction negatively regulates T cell activation, cytotoxicity, and cytokine production. In fact, the LAG-3-Ig fusion protein competes for binding in CD4/MHC-II-dependent cell adhesion assays. Once LAG-3 binds to MHC-II, it transmits inhibitory signals through its cytoplasmic domain, thereby inhibiting the activation of CD4+ T cells (142).

The second identified ligand of LAG-3 is Galectin-3 (Gal-3), a soluble lectin that binds to galactosides and has a molecular weight of approximately 31 kDa. Gal-3 regulates T cell activation and is highly expressed in various tumor cells and activated T lymphocytes (83). The interaction between Gal-3 and LAG-3 is essential for optimal inhibition of CD8+ T cell cytotoxicity (143). Within the tumor microenvironment, Gal-3, via LAG-3 expression, inhibits the activation of antigen-specific CD8+ T cells and suppresses the expansion of plasmacytoid DCs, thereby impeding the formation of an effective anti-tumor immune response (144).

Fibrinogen-like protein 1 (FGL1), secreted by the liver, has recently been identified as a functional ligand for LAG-3 (145). FGL1 binds to the D1 and D2 domains of LAG-3, and while a single point mutation (Y73F) in the D1 domain disrupts MHC-II binding, it does not affect FGL1-Ig binding. This suggests that FGL1 and LAG-3 interact independently of MHC-II (146). FGL1 expression is induced by IL-6 and is present at low levels in the liver but highly upregulated in certain human cancers, such as lung cancer, melanoma, anterior adenocarcinoma, and colorectal cancer in the United States. FGL1 exhibits high affinity for LAG-3, and their interaction facilitates tumor immune escape. Blocking the FGL1-LAG-3 pathway has been shown to enhance the anti-tumor activity of CD8+ T cells (147).

In addition to FGL1, several other ligands for LAG-3 have been identified. One such ligand is LSECtin (liver sinusoidal endothelial cell lectin), a member of the C-type lectin receptor superfamily and a type II transmembrane protein. It is highly expressed in the liver and melanoma cells, where it inhibits the immune responses of CD8+ T cells and NK cells through its interaction with LAG-3. Another ligand, α-Synuclein, like MHC-II, binds to the LAG-3 D1 region and relies on the D2, D3, or intracellular domains (148).

## PVR family

The poliovirus receptor (PVR) family, a group of proteins associated with immune regulation, belongs to the IgSF (149). Initially referred to as the PVR-related Ig domain (PVRIG) due to its inclusion of an Ig domain, this family comprises multiple members, including T cell immunoglobulin and immune receptor tyrosine inhibitory motif domains (TIGIT), CD96, CD226, as well as their ligands CD155 and CD112 (150).

Members of the PVR family share structural homology and exert synergistic or inhibitory effects through highly interactive interactions, forming a complex immune regulatory network (151). These proteins are of significant importance in immunotherapy, particularly in the treatment of hematological malignancies, making them a focal point of research.

PVR/nectin family members are expressed on various lymphocytes, including NK cells, CD8+, CD4+, and Tregs. TIGIT, DNAM-1 (CD226), CD96, and CD112R are expressed on T cells and natural killer (NK) cells, while their ligands—CD155, CD112, CD113, and CD111—are expressed on APCs or tumor cells (152). NK cells play a crucial role in eliminating and preventing metastasis during the early stages of cancer. As cytolytic effector cells, NK cells are involved in the release of tumor antigens, and the regulation of NK cell function by TIGIT significantly impacts the initial phase of the cancer immune cycle (153).

TIGIT, CD155 (PVR), CD96, CD226, and other related proteins share structural similarities and are collectively known as the CD155 family (154). Unlike typical immune checkpoint-ligand interactions, which generally follow a one-to-one or one-to-many relationship, TIGIT maintains a “many-to-many” relationship with CD226, CD96, CD112, and CD155 (155). This positions TIGIT within a complex regulatory network that includes multiple receptors (such as CD96 and CD112R), a competitive co-stimulatory receptor (CD226), and multiple ligands (such as CD155 and CD112) (156). This network is somewhat analogous to the CD28/CTLA-4/CD80/CD86 pathway, where inhibitory and co-stimulatory receptors compete for binding to the same ligands (157).

### TIGIT

TIGIT (T-cell immunoreceptor with Ig and ITIM domains) (also known as WUCAM, Vstm3, VSIG9) is a member of the PVR/adhesin family, which belongs to the IgSF (158). It consists of an extracellular immunoglobulin variable region (IgV) domain, a type 1 transmembrane domain, and an intracellular domain with a classical immune receptor tyrosine inhibitory motif (ITIM) and immunoglobulin tyrosine tail (ITT) motif (159). TIGIT was initially discovered in a gene study on T cell specific expression by Genentech’s research team. The TIGIT gene is located on chromosome 3q13.31 and encodes a protein with 244 amino acids (160, 161).

TIGIT has been reported as a marker of CD8+T cell failure and a characteristic marker of Tregs in the tumor microenvironment (162). Another notable feature of TIGIT is that it is N-linked glycosylation, which often occurs on the asparagine residue in the N-X-S/T glycosylation sequence. N is asparagine, X is any amino acid except proline, S is serine, and T is threonine. N-glycosylation involves many aspects of cell biology, such as intercellular information transmission, ligand/receptor interactions, and cellular signal transduction. A study on PD-1 suggests that the interaction between immunosuppressive ligands/receptors is also widely dependent on n-glycosylation (163). In order to investigate whether the n-glycosylation of TIGIT is crucial for its ligand binding activity, a study combined TIGIT deglycosylation with *in vitro* PVR/TIGIT binding experiments. It was found that eliminating n-glycans from TIGIT inhibited the binding of TIGIT to PVR, indicating that the n-glycosylation of TIGIT is crucial for the involvement of PVR/TIGIT (160).

TIGIT is thought to compete with co-stimulatory receptors CD226 (also known as DNAM-1) and CD96 on T cells for binding to ligands such as CD155, CD112, and CD113 (164). The primary ligand for TIGIT is CD155, though immunoprecipitation experiments have demonstrated that CD112 and CD113 can also weakly interact with TIGIT. The IgV domain of TIGIT contains unique motifs, including (V/I)(S/T)Q, AX6G, and T(F/Y)PX1G subunits, which are involved in mediating trans interactions with PVR family cis dimers (165). These conserved motifs are characteristic of the PVR/nectin family, which includes TIGIT, CD226, CD96, CD112R, PVR, CD112, and CD113 (also known as PVRL3/nectin3) (166).

In mice, phosphorylation of the ITIM (Y227) or ITT-like motif residue (Y233) can trigger TIGIT-mediated inhibitory signaling (30). However, in the human NK cell line YTS, TIGIT/CD155 interaction predominantly initiates inhibitory signaling through the ITT-like motif. Upon TIGIT/CD155 engagement, phosphorylation of Tyr225 within the ITT-like motif occurs, facilitating the recruitment of cytoplasmic signaling molecules Grb2 and β-arrestin 2, which subsequently recruit the inositol-containing SH2 phosphatase-1 (SHIP-1). SHIP-1 inhibits the activation of PI3K and MAPK pathways while also suppressing TRAF6 and NF-κB signaling, leading to reduced IFN-γ production by NK cells (167). Moreover, TIGIT binding to DCs induces CD155 phosphorylation and activates a signaling cascade that promotes the formation of tolerogenic DCs, characterized by decreased IL-12 production and increased IL-10 secretion (168).

**Figure 4:**
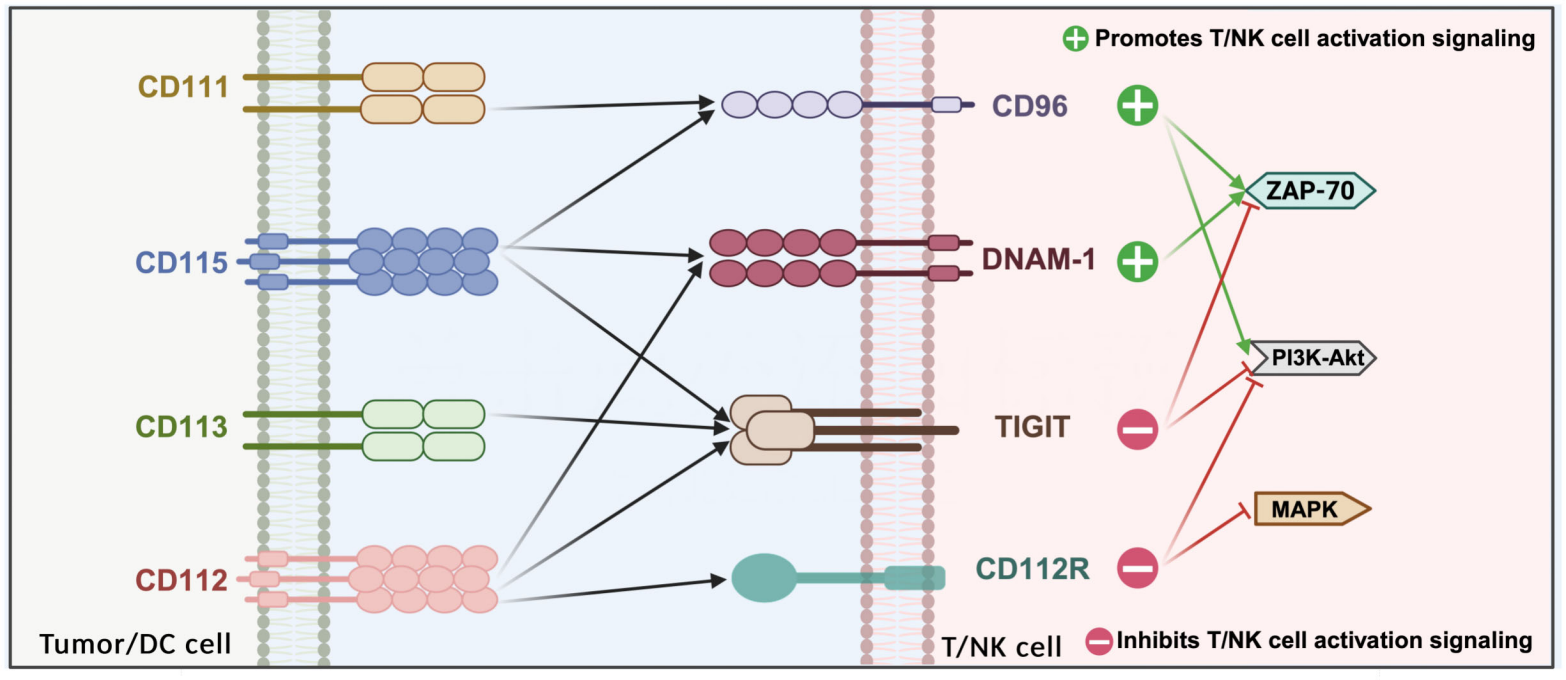
Interactions between the various members of the PVR family.

Recently, Nectin-4 has been identified as a novel ligand for TIGIT. Nectin-4 binds to TIGIT with an affinity similar to that of CD155 but uniquely does not interact with CD226, CD96, or CD112 (169). TIGIT, DNAM-1, CD96, and CD112R are expressed on T cells and natural killer (NK) cells, while their respective ligands—CD155, CD112, CD113, and CD111—are expressed on APCs or tumor cells. CD155 is predominantly expressed on DCs, T cells, B cells, and macrophages, whereas CD112 is broadly expressed in both hematopoietic and non-hematopoietic tissues, including bone marrow, lungs, pancreas, and kidneys. In contrast, CD113 expression is restricted to non-hematopoietic tissues, such as the lungs, liver, testes, kidneys, and fetal tissues (170) (**Figure 4**).

TIGIT, CD112R, and CD155 transmit inhibitory signals to cells through their cytoplasmic tails, whereas DNAM-1 continues to transmit activation signals. The crystal structure of TIGIT bound to CD155 reveals that two TIGIT/CD155 dimers assemble into a heterotetramer with a core TIGIT/TIGIT cis homodimer, where each TIGIT molecule binds to a CD155 molecule. This cis-trans receptor aggregation mediates cell adhesion and signal transduction (171). TIGIT effectively inhibits both innate and adaptive immunity through various mechanisms. Antibodies that competitively bind to TIGIT can directly inhibit T cell proliferation and function by attenuating TCR-driven activation signals. Moreover, TIGIT binding induces the phosphorylation of CD155 in DCs, triggering a signaling cascade that reduces the expression of interleukin-12 and interleukin-10 in tolerogenic DCs, thereby indirectly impairing T cell function. Concurrently, TIGIT inhibits NK cell degranulation, cytokine production, and the cytotoxicity of NK cells against tumor cells expressing CD155. By competing with CD155 with high affinity, TIGIT hinders CD155-mediated activation of CD226. In CD226-deficient mouse models, CD8+ T cells and NK cells exhibit defects in immune synapse formation, which impairs their anti-tumor immune functions (172, 173).

TIGIT also presents a safety advantage in therapeutic applications. The interaction of TIGIT on Tregs disrupts cytokine balance, inhibits Th1 or Th17 phenotypes, and induces Th2 phenotypes. However, unlike CTLA-4 and PD-1, TIGIT knockout in mice does not result in a severe spontaneous autoimmune phenotype, suggesting that TIGIT moderates the immune response without triggering severe autoimmunity (174).

Currently, targeting the TIGIT-PVR pathway is gaining importance, with several biotechnology and pharmaceutical companies developing antibodies or dual antibodies against TIGIT that are at various stages of clinical development. Globally, major pharmaceutical companies such as Roche, Bristol-Myers Squibb, and MSD are leading the way, with Roche and MSD having made the most progress, both being in Phase III clinical trials. Meanwhile, additional immune checkpoint inhibitors have exhibited promising efficacy across a diverse spectrum of cancers, with ongoing research into novel checkpoint molecules and combination therapies advancing at a rapid pace. Nevertheless, challenges such as drug resistance and immune-related adverse effects remain significant barriers in the development process. Future studies are therefore expected to focus on refining drug efficacy and safety profiles to facilitate broader and more effective clinical applications (**Table 4**).

**Table 4:** Summary of immune checkpoint monoclonal antibody drugs and their pathways of action.

Medicine	R&D company	Drug properties and effects	Development phase	References
VISTA				
CI-8993 (NCT04475523)	Curis	CI-8993 is a monoclonal IgG1 with active Fc that antagonizes VISTA κ Antibodies. CI-8993 as a monotherapy can inhibit the growth of transplantable and inducible melanoma.	Phase I clinical trial	(175)
HMBD-002 (NCT05082610)	Humminbird Bioscience	HMB-002 is an IgG4 type antagonistic monoclonal antibody against VISTA that does not rely on Fc. It was developed under the guidance of AI and targets a conserved specific functional epitope on the C-C ‘ring specific to VISTA. It has shown effective inhibition of tumor growth in humanized mouse cancer models of preclinical colorectal cancer, lung cancer and breast cancer.	Phase I clinical trial	(176)
W0180 (NCT04564417)	Pierre Fabre medical care	W0180 is a monoclonal antibody targeting VISTA. *In vitro* experiments have shown that W0180 stimulates NK cell proliferation and induces the activation of cytokines by NK cells and monocytes, promoting T cell activation.	Phase I clinical trial	(177)
CA-170 (NCT02812875)	Curis	CA-170 is an oral small molecule peptide dual antagonist that selectively targets PD-L1 and VISTA. CA-170 has shown good safety and efficacy in the treatment of various types of tumors, including head and neck squamous cell carcinoma, NSCLC, MSI-H positive solid tumors, and Hodgkin’s lymphoma.	Phase II/III clinical trial	(178)
SG7	Stanford University	SG7 is an antagonistic VISTA antibody designed and constructed using yeast surface display. In mouse experiments, SG7 can be used in combination with anti-PD1 to slow down tumor growth in various homologous mouse models.	Preclinical experiments	(179)
P1-068767 (BMS-767)	Bristol Myers Squibb	BMS-767 is an antagonistic VISTA monoclonal antibody that selectively blocks the interaction between PSGL-1 and VISTA at pH 6.0, potentially reducing any non-tumor reactivity and adverse effects.	Preclinical experiments	(180)
B7-H3				
Ifinatamab Deruxtecan (NCT06330064)	Daiichi Sankyo	As of January 31, 2023, among 21 small cell lung cancer (SCLC) patients, the ORR was 52%, including 1 complete response (CR) and 10 partial responses (PRs). The median duration of response (DOR) was 5.9 months (95% CI, 2.8–7.5), the median progression-free survival (PFS) was 5.8 months (95% CI, 3.9–8.1), and the median OS was 9.9 months (95% CI, 5.8–not reached).	Phase II clinical trial	(181)
HS-20093 (NCT05830123)	Hansoh BioMedical R&D Company	Among 40 advanced pretreated solid tumor patients, the ORR was 35%, and the disease control rate (DCR) reached 85%, regardless of baseline B7-H3 expression levels. In 11 evaluable small cell lung cancer (SCLC) patients, the ORR was 63.6%, with all responses observed at the first disease assessment and a median time to response of 6 weeks. The DCR was 81.8%, with a median progression-free survival (PFS) of 4.7 months and a 3-month PFS rate of 72.7%.	Phase I clinical trial	(182)
LAG-3				
Relatlimab (NCT03607890)	Bristol Myers Squibb	Used to treat adult and pediatric patients aged 12 years or older with unresectable or metastatic melanoma, and for the treatment of NSCLC, HCC, and colorectal cancer	FDA approved	(183)
Favezelimab (NCT02720068)	Merck&Co	Favezelimab (MK-4280) is a humanized anti-LAG-3 monoclonal antibody that can block the interaction between LAG-3 and its ligand MHC class II. Favezelimab has the potential to be used in combination with the PD-L1 inhibitor Pembrolizumab (HY-P9902) for research on colorectal cancer (CRC).	Phase I clinical trial	(184)
Ieramilimab (NCT03484923)	Pierre Fabre medical care	Iramilimab (LAG525; IMP701) is a humanized IgG4 monoclonal antibody that can bind to LAG-3, thereby inhibiting the interaction between LAG-3 and MHC-II molecules.	Phase I clinical trial	(185)
TIGIT				
Tiragolumab (NCT05798663)	Roche	Tiragolumab is undergoing multiple clinical trials, mainly targeting various solid tumors such as NSCLC, melanoma, gastric cancer, and esophageal cancer.	Phase III clinical trial	(186)
Vibostolimab (NCT05005442)	Merck & Co	Vibostolimab binds to TIGIT and blocks the interaction between TIGIT and its ligands (CD112 and CD155). Activation helps T lymphocytes destroy tumor cells and can be used for the treatment of NSCLC and melanoma.	Phase III clinical trial	(187)
AB154 (NCT04656535)	Arcus	AB154(Domvanalimab) is a monoclonal antibody targeting TIGIT. Domvanalimab blocks the binding of CD155 on the surface of cancer cells to TIGIT on the surface of immune cells, causing CD155 to bind to DNAM-1 protein and activate the immune signaling pathway. Clinical trials have focused on the combination therapy with PD-1 monoclonal antibody zimberelimab, mainly targeting NSCLC.	Phase II clinical trial	(188)
TIM-3				
Sabatolimab (NCT04623216)	Novartis	Sabatolima targets the TIM-3 receptor. This receptor is mostly expressed on the surface of immune cells and myeloid leukemia cells, and can innovatively target both myeloid leukemia cells and immune cells, which not only kills cancer cells, but may also enhance the viability of immune cells.	Phase III clinical trial	(189)
Cobolimab (NCT06521567)	Tesaro	Cobolimab was the first anti-TIM-3 drug to publish trial data, a humanized anti-TIM3 IgG4 antibody developed by Tesaro.Cobolimab+dostarlimab was well tolerated and showed preliminary antitumor activity.	Phase III clinical trial	(190)
SIRPα				
BI-765063 (NCT04653142)	Boehringer Ingelheim & OSE Immunotherapeutics	BI 765063 prevents ligand binding between SIRPα and CD47 by binding to SIRPα, thereby blocking cellular signaling that would lead to a decrease in anti-tumor substances (e.g., macrophages and DCs) in myeloid cells.	Phase I clinical trial	(191)
CC-95251 (NCT05168202)	Celgene & BSM	CC-95251 is used in the treatment of hematologic tumors to reduce neutrophil infiltration and has demonstrated a favorable safety and efficacy profile in the treatment of these tumors.	Phase I clinical trial	(192)
AL008 (NCT01243242)	Innovent Biologics	IBI397 is a dual-mechanism inhibitor. Instead of directly blocking the binding of SIRPα to CD47, IBI397 blocks SIRPα-CD47 pathway signaling by mediating endocytosis of SIRPα on macrophages; in addition, the Fc-terminal end of IBI397 binds to the activated FcγR, which further enhances the tumor immunity and achieves the purpose of tumor suppression.	Preclinical trial	(193)
OX-40				
PF-04518600 (NCT03092856)	Pfizer	PF-04518600 selectively binds and activates OX40 to induce proliferation of memory and effector T lymphocytes. In the presence of tumor-associated antigen (TAA), this may promote T cell-mediated immune responses against TAA-expressing tumor cells. Indications targeted are metastatic renal cancer, triple-negative breast cancer, and advanced malignancies, respectively.	Phase II clinical trial	(194)
IBI101 (NCT03758001)	Innovent Biologics	IBI101 is an OX40 agonist intended for the treatment of a variety of solid tumor diseases. Data from preclinical studies confirm that IBI101 has a well-defined mechanism of action, which significantly enhances the activation of effector T cells and mediates the clearance of tregs, thereby acting to inhibit the growth of tumor cells.	Phase I clinical trial	(195)
GBR830(**OX40 Inhibitor Antibody)** (NCT0268392)	Glenmark Pharmaceutical	GBR830 inhibits the binding of OX40 and OX40L in activated T cells and tregs, potentially reducing inflammation associated with atopic dermatitis symptoms.	Phase II clinical trial	(196)
Rocatinlimab(**OX40 Inhibitor Antibody)** (NCT06438263)	Amgen Inc.	Rocatinlimab is an OX40 agonist for the treatment of moderate to severe atopic dermatitis (AD) and is currently undergoing a multicenter, double-blind maintenance study of long-term safety, tolerability and efficacy in adult and adolescent subjects.	Phase III clinical trial	(197)
4-1BB				
Urelumab (NCT01471210)	BMS	Urelumab was the first targeted 4-1BB therapy to enter clinical trials, and it is an IgG4 monoclonal antibody. Previous experimental data showed liver toxicity. Urelumab was re-entered into clinical trials in 2012, and studies are currently underway to investigate the potential of Urelumab in combination with other drugs for the treatment of solid tumors such as glioblastoma and pancreatic cancer.	Phase II clinical trial	(198)
Utomilumab (NCT03258008)	Pfizer	It is a 4-1BB humanized IgG2 monoclonal antibody developed by Pfizer, which has a higher safety profile relative to urelumab and is also currently in multiple clinical trials, but is a less potent 4-1BB agonist relative to urelumab.	Phase III clinical trial	(199)

## TIM family

In humans, the TIM family includes TIM1, TIM3, and TIM4, located on chromosome 5q33.2. In mice, the TIM family includes TIM1 to TIM8, located on chromosome 11B1.1. TIM proteins are a class of transmembrane glycoproteins characterized by a common motif. Their structure comprises five regions: signal peptide, immunoglobulin, mucin, transmembrane, and intracellular tail (200). Except for TIM-4, the intracellular regions of TIM-1, TIM-2, and TIM-3 contain tyrosine phosphorylation motifs that participate in transmembrane signal transduction.

The TIM (T cell/transmembrane, immunoglobulin, and mucin) gene family proteins first garnered attention in virology due to their phosphatidylserine (PtdSer) receptor epitopes, which play a crucial role in enhancing viral entry (201). Subsequently, substantial data has accumulated indicating that this gene family is pivotal in regulating immune responses, including transplant immune tolerance, autoimmunity, allergies, and asthma (202).

The TIM proteins may function as a novel receptor family for phosphatidylserine (PtdSer), binding to this key “Eat me” signaling molecule, mediating the phagocytic clearance of apoptotic cells, and playing a crucial role in regulating immune tolerance *in vivo* while maintaining internal homeostasis (203). The unique structure of the TIM immunoglobulin variable domain enables highly specific recognition of PtdSer exposed on the surface of apoptotic cells. The crystal structures of Tim-1, Tim-2, Tim-3, and Tim-4 in rodents reveal a characteristic FG-CC’ motif (204). While TIM-1, TIM-3, and TIM-4 can recognize PtdSer, their expression on different cells suggests distinct functions in immune regulation. Consequently, the TIM gene family is essential for immune response and tolerance. Research has demonstrated that the PS receptor TIM-4 regulates adaptive immune responses *in vivo* by mediating the antigen-specific clearance of apoptotic T cells (205).

TIM-1 is a significant susceptibility gene for asthma and allergy, preferentially expressed on T helper cell 2 (Th2) cells, and serves as an effective co-stimulatory molecule for T cell activation. TIM-3, expressed on the surface of Th1 cells, binds to its ligand galectin-9. Through the TIM-3-galectin-9 binding pathway, it generates inhibitory signals, induces Th1 cell death, and negatively regulates the Th1 immune response (206). It has been found that TIM-4 expressed by APCs is a ligand for TIM-1. *In vivo* injection of either soluble TIM-1 immunoglobulin (TIM-1-Ig) fusion proteins or TIM-4-Ig fusion proteins resulted in T-cell over proliferation, and TIM-4-Ig stimulated CD3- and CD28-mediated T-cell proliferation *in vitro*. These data suggest that TIM-1-TIM-4 interaction is involved in the regulation of T cell proliferation (207).

### TIM-1

The T cell immunoglobulin and mucin (TIM) family plays a critical role in regulating T cell-mediated immune responses. Among its members, TIM-1 is notably involved in modulating Th1/Th2 cell differentiation (208). The TIM-1 gene, identified on mouse chromosome 11, has been shown to confer protection against Th2-mediated airway hyperresponsiveness, making it a valuable focus of asthma research. Beyond its association with airway hyperresponsiveness, TIM-1 is predominantly expressed by Th2 cells, further underscoring its significance in Th2-driven immune processes (209). Additionally, TIM-1 signaling was found to influence antibody production both *in vitro* and *in vivo*, with higher levels of IgG2b and IgG3 detected in the culture supernatants of anti-TIM-1-stimulated B cells. When immunized with the T-independent antigen TNP-Ficoll, TNP-specific IgG1, IgG2b, and IgG3 antibodies were slightly increased in anti-TIM-1-treated mice (210).

In 2023, a team from Harvard Medical School identified TIM-1 as a critical immune checkpoint in B cells and investigated strategies to bypass this checkpoint to enhance the anti-tumor potential of T cells. Targeting TIM-1 to inhibit B cells can amplify anti-tumor CD8+ and CD4+ T-cell responses and suppress tumor growth. This study identifies TIM-1 as a pivotal immune checkpoint for B-cell activation. TIM-1 modulates the type 1 interferon (IFN-1) response in B cells, thereby limiting B-cell activation, antigen presentation, and co-stimulation, which underscores TIM-1 as a potential target for enhancing B-cell-mediated anti-tumor immunity (35).Given that TIM-4 is a homologous ligand of TIM-1, it is insightful to consider the role of TIM-1 in promoting T-cell expansion and survival via its interaction with TIM-4, suggesting that the TIM-1 pathway serves as a natural stimulator of T-cell function (211).

### TIM-3

T-cell immunoglobulin mucin 3 (TIM-3), also known as HAVCR2, is a critical tumor immune checkpoint that was first identified in 2002,TIM-3 functions as a negatively regulated immune checkpoint. The TIM-3 gene is located on chromosome 5q33.2, encodes a protein comprising 281 amino acids, and consists of an extracellular region, a single transmembrane structural domain, and a C-terminal cytoplasmic tail (212). TIM-3 is a class of inhibitory molecules found on the surface of T cells, which contribute to T-cell exhaustion in the context of cancer and chronic viral infections. Similar to PD-1 and CTLA-4, TIM-3 is one of the most extensively studied targets for immunotherapy. It has been observed that patients treated with anti-PD-1 or anti-PD-L1 monoclonal antibodies often develop resistance, and TIM-3 expression is upregulated in response to adaptive resistance to anti-PD-1 therapy (213).

TIM-3 is selectively expressed on IFN-γ-secreting helper T cells (Th1 and Th17), Tregs, mast cells, DCs, NK cells, tumor-infiltrating lymphocytes (TILs), monocytes, as well as on tumor cells such as melanoma, gastric cancer, and B-cell lymphoma (214).

The mechanism by which TIM-3 functions as a crucial immune checkpoint is primarily due to its identification of the most dysfunctional subpopulation of tumor-infiltrating CD8+ PD-1+ T cells (215). Antibodies that simultaneously block the TIM-3 and PD-1 pathways exhibit a synergistic effect, enhancing tumor growth inhibition and improving the response of tumor antigen-specific CD8+ T cells (216).

**Figure 5:**
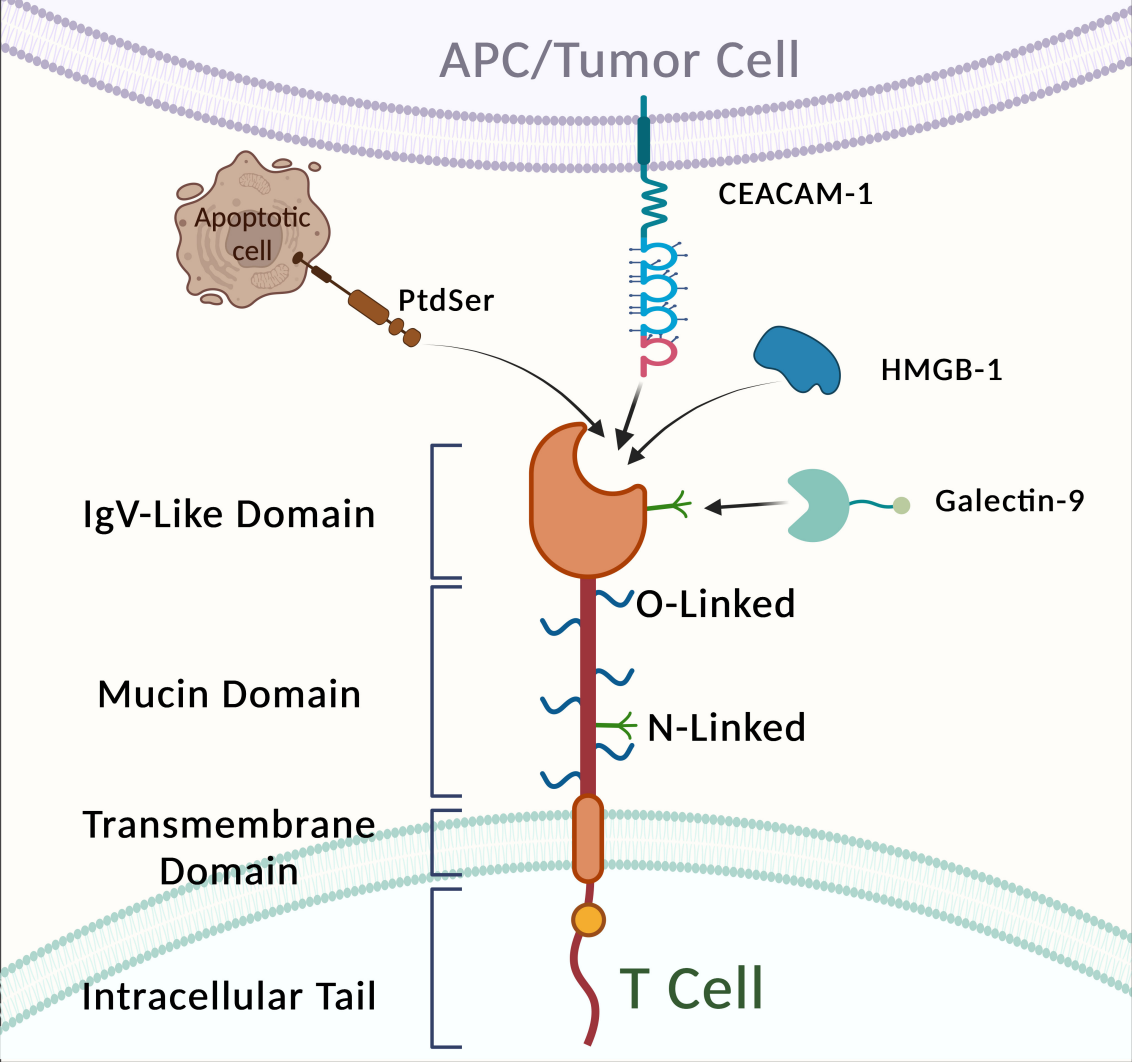
Structure and ligands of TIM-3.

Transcriptomic analysis revealed a significant enrichment of the PI3K-AKT and MAPK signaling pathways in TIM-3 knockout (KO) tumor cells compared to TIM-3^+^ tumor cells. Furthermore, evaluation of an anti-TIM-3 monoclonal antibody demonstrated its efficacy in significantly prolonging the survival of DIPG mice (217).This chromosomal region has been consistently associated with asthma, allergies, and autoimmune diseases. TIM proteins are a class of transmembrane glycoproteins characterized by a common motif, with a structure comprising five regions: a signal peptide, an immunoglobulin region, a mucin region, a transmembrane region, and an intracellular tail (**Figure 5**).

While TIM-3-expressing fibroblasts and APCs are involved in the phagocytosis of apoptotic cells, TIM-3-expressing T cells bind to but do not phagocytose these cells. These observations suggest that TIM-3-expressing DCs, macrophages, and T cells are capable of detecting apoptotic cells (218). TIM-3 has four known ligands: galectin-9 (Gal-9), carcinoembryonic antigen cell adhesion molecule-1 (CEACAM-1), high mobility group protein B1 (HMGB1), and phosphatidylserine (PS) (219). Gal-9, the first ligand identified, is a carbohydrate-binding protein that recognizes N-linked glycans in the TIM-3 IgV domain. The interaction between TIM-3 and Gal-9 inhibits tumor immunity by suppressing T-cell activity, effectively halting Th1 immune responses through binding to the TIM-3 IgV domain (220).

Recent findings indicate that elevated TIM-3 expression is observed on CD4+ and CD8+ T cells in the peripheral blood of patients with acute hepatitis B (AHB) and chronic hepatitis B (CHB) (221). Furthermore, an increase in TIM-3+ T cells correlates positively with conventional liver injury markers, including alanine aminotransferase (ALT), aspartate aminotransferase (AST), total bilirubin (TB), and the international normalized ratio (INR). Conversely, TIM-3 expression is negatively correlated with T-bet mRNA expression and plasma interferon-gamma (IFN-γ) levels. These results suggest that TIM-3 overexpression is involved in CHB disease progression and may contribute to the skewed Th1/Tc1 response that leads to persistent HBV infection.

HCV(hepatitis C virus) evades host immune attack and apoptosis through various mechanisms, including the production of quasispecies, viral-specific and general immunosuppression, Tregs, and induction of PD-1/TIM-3-mediated exhaustion in effector T cells (Teff) (222). TIM-3 may play a significant role in the natural immune response by interacting with the negative regulators Programmed Death-1 (PD-1) and Suppressor of Cytokine Signaling-1 (SOCS-1) (223). This interaction inhibits STAT-1 phosphorylation and negatively regulates the production of interleukin-12 (IL-12), suggesting that TIM-3 may serve as a crucial target for HCV treatment (224).

TIM-3 is among the most extensively researched targets in immunotherapy. However, no TIM-3-targeted drugs are currently approved or marketed globally. Novartis and GSK are advancing TIM-3 inhibitors through Phase III clinical trials, while Roche and Bajaj Shenzhou are conducting Phase II trials. In China, Hengrui and Zhikang Hongyi are in Phase I clinical trials. Additionally, Fuhong Hanklin, Vannes, Zhao Derivatives, Zhiren Meibao, and Lizumab are at the preclinical stage. TIM-3 remains a prominent focus in immunotherapy research, with no TIM-3-targeted drugs yet listed. Novartis and GSK are in Phase III trials, Roche and Bajaj Shenzhou are in Phase II, and AZD7789, a key PD-1/TIM-3 bispecific monoclonal antibody developed by AstraZeneca, is set to enter clinical trials in the U.S. in 2021. This antibody targets advanced solid tumors and hematological malignancies. This led us to summarize multiple immunotherapeutic agents with immune checkpoints that have similar bidirectional specificity to AZD7789 (**Table 5**).

**Table 5:** Summary of immune checkpoint bispecific antibody drugs and their pathways of action.

Medicine	R&D company	Drug properties and effects	Development phase	References
LAG-3&PD-1				
Tebotelimab (NCT03219268)	MacroGenics	Tebotelimab is a PD-1/LAG-3 bispecific tetravalent DART molecule developed by Zaiding Pharmaceuticals for the treatment of advanced mucosal melanoma patients treated on the first line. In preclinical studies, it has been shown to have synergistic anti-tumor activity.	PreclinicaII-III experiments	(225)
EMB-02 (NCT04618393)	EpimAbBiotherapeutics	EMB-02 is a symmetric IgG like bispecific antibody targeting human programmed cell death protein 1 (PD-1) and lymphocyte activation gene 3 (LAG-3), based on FIT-Ig ^®^ Developed through technology for the treatment of advanced solid tumors.	PreclinicaII experiments	(226)
TIGIT&PD-1				
MK-7684A (NCT05224141)	Merck & Co.	MK-7684A is a fixed dose compound formulation composed of Merck Vibostolimab (MK-7684) and Pembrolizumab (K-drug), which can block the interaction between TIGIT/PD-1 and its ligand, thereby activating T lymphocytes and enhancing the attacking ability of tumor cells.	Preclinica II experiments	(227)
BMS-986442 (NCT05543629)	Agenus & Bristol-Myers Squibb	BMS-986442 has an enhanced Fc region that can improve tumor responsive T cell response. In order to achieve better activation of T cells or NK cells, it is being developed for use in NSCLC and gastric cancer.	Phase II clinical trial	(228)
TIM-3&PD-1				
AZD7789 (NCT04931654)	AstraZeneca	AZD7789 is AstraZeneca’s key investigational PD-1/TIM-3 bispecific monoclonal antibody, which will be the first to enter the clinic in the U.S. in 2021, with indications for advanced solid tumors and hematologic malignancies.	Phase II clinical trial	(229)

## SIRP family

Signal regulatory proteins (SIRPs) are a family of cell surface signaling receptors, consisting of five members: SIRPα, SIRPβ1, SIRPγ, SIRPβ2, and SIRPδ (230). These receptors are differentially expressed in leukocytes and the central nervous system, with predominant expression on the surface of myeloid cells, such as monocytes, macrophages, granulocytes, and myeloid DCs in humans (230). SIRPs are also expressed in certain cancer cells and neuronal cells of the nervous system, of all the members. SIRPα is notable for being the immune checkpoint protein with the strongest binding affinity to CD47 (231).

Structurally, SIRPs belong to the IgSF, characterized by an N-terminal extracellular domain containing three cysteine-binding Ig-like loops, a single transmembrane domain, and a C-terminal intracellular domain (232). The C-terminal intracellular domain of the SIRPα subfamily contains a relatively long amino acid sequence (110 amino acids in SIRPα) that includes four tyrosine residues, which form two immunoreceptor tyrosine-based inhibitory motifs (ITIMs) (233).

### SIRPa

SIRPα (also known as PTPNS1, SHPS-1, CD172a, and P84) is known for binding to CD47. Signal regulatory protein alpha (SIRPα) is a transmembrane protein whose extracellular region consists of three Ig-like structural domains and a cytoplasmic region containing immunoreceptor tyrosine-based inhibitory motifs (ITIMs) that mediate binding of the protein tyrosine phosphatases SHP-1 and SHP-2 (234). SIRPα is particularly abundant in myeloid cells such as macrophages and DCs (235), with lower expression levels in T cells, B cells, NK cells, and NKT cells. Polymorphic allelic variants in the ligand-binding domain have been reported in African, Japanese, Chinese, and Caucasian populations, with three of them (SIRPαV1, SIRPαV2, and SIRPαV8) being the most prominent haplotypes, covering about 90% of the population (236).

SIRPα inhibits macrophage phagocytosis by interacting with its ligand, CD47, a key immunosuppressive signaling molecule involved in the immune escape of tumor cells. CD47 is typically upregulated on the surface of malignant cells, sending a “don’t-eat-me” signal to immune cells, helping to maintain immune tolerance in non-malignant cells under physiological conditions (237). However, this mechanism can also enable cancer cells to survive in various types of cancer. In many cancer types, CD47, which binds to signal-regulatory protein alpha (SIRPα), initiates inhibitory signaling pathways that prevent malignant cells from being phagocytosed by macrophages (238).

A 2022 study from Texas MD Anderson Cancer Center highlighted the dual role of SIRPα in cancer treatment. Analysis of 60 immuno-oncology genes in melanoma patients revealed that higher SIRPα expression in tumor cells correlated with better responses to anti-PD-1 therapy and improved patient outcomes, contrasting with its traditional immunosuppressive role in macrophages. Single-cell proteomics confirmed that elevated SIRPα expression originated from melanoma cells rather than macrophages and enhanced T cell-mediated tumor killing. These findings suggest tumor cell-expressed SIRPα enhances sensitivity to immunotherapy, while macrophage-expressed SIRPα maintains its inhibitory role. Additionally, SIRPα-targeting antibodies show promise as safer immunotherapy agents, requiring low doses to block CD47-SIRPα interactions without significant hematological side effects (239).

**Figure 6:**
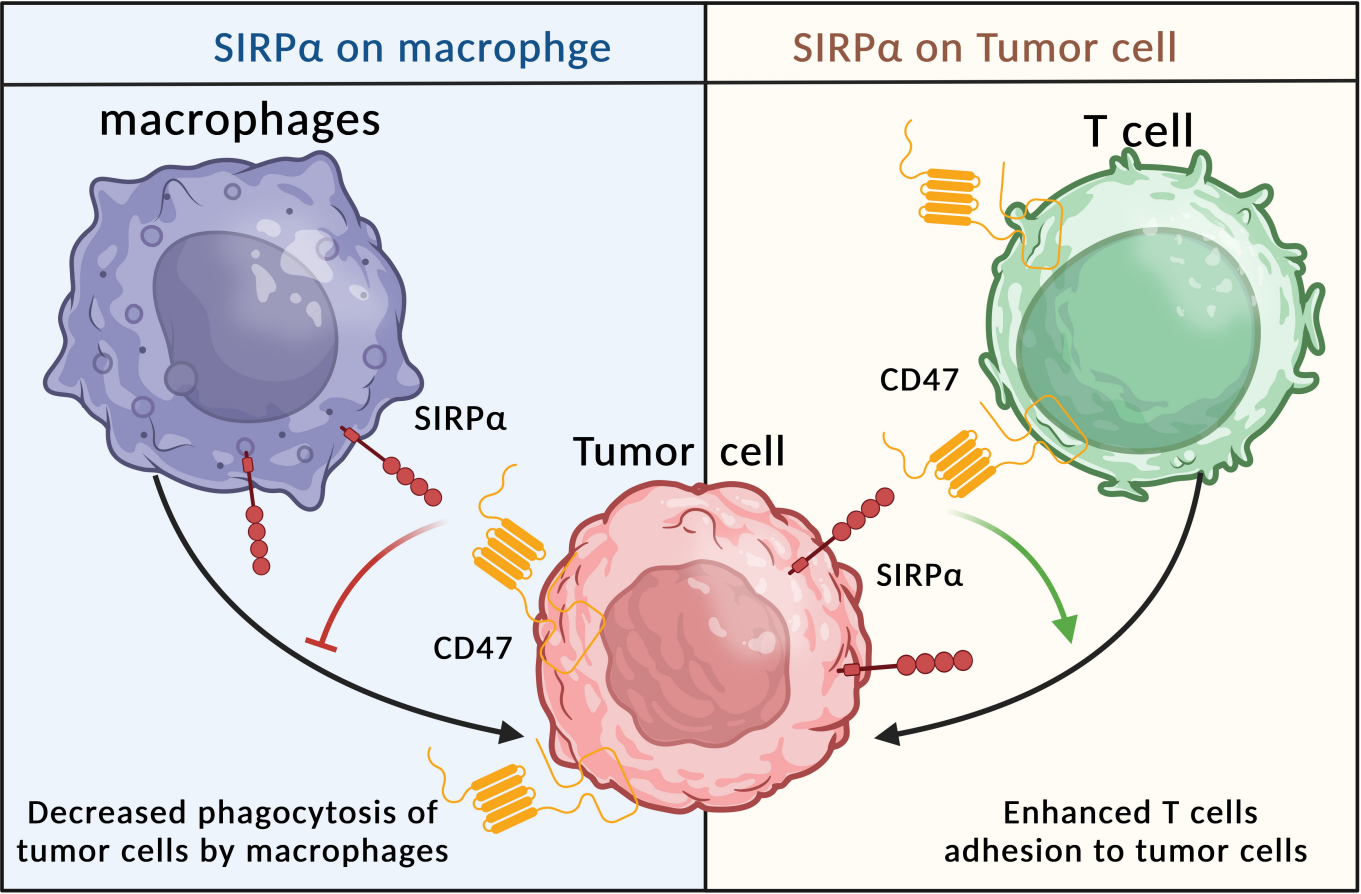
Immune responses regulated by SIRPα expression on different cells.

The above studies have shown that the same target in different cell types can have different effects on immunotherapy, thus positioning SIRPα as a promising target with dual immune effects (**Figure 6**).

SIRPα-targeting antibodies are considered safer because SIRPα is primarily expressed on myeloid cells. A small dose of SIRPα antibody is sufficient to block the CD47-SIRPα pathway in tumor cells without leading to erythrocyte destruction or other hematological adverse effects. This distinction makes SIRPα-targeting antibodies a potentially safer alternative in cancer immunotherapy (240).

## TNFSF family

The Tumor Necrosis Factor Superfamily (TNFSF) consists of proteins that share TNF homology domains at the C-terminus and form a trimeric structure (241). TNFSF ligands can bind to members of the Tumor Necrosis Factor Receptor Superfamily (TNFRSF), thereby regulating a variety of cellular processes, including immune responses, inflammation, and cell proliferation, differentiation, and apoptosis (242). The TNFSF/TNFRSF system includes 19 ligands and 29 receptors, with some ligands capable of binding to multiple receptors and some receptors interacting with more than one ligand. This ligand-receptor sharing creates an extensive communication network that facilitates the regulation of complex cellular responses (243).

When TNFRSF binds to its ligands, the resulting interaction can regulate cell survival and function through activation of the NF-κB or MAPK pathways via TNFR-associated factors (TRAFs) (244). Conversely, binding of TNFRSF to ligands containing death domains can ultimately lead to the activation of caspases and programmed cell death (245). Another subgroup within TNFRSF, such as CD137, glucocorticoid-induced TNF receptor (GITR), and OX40, activate NF-κB, promoting cell survival (246).

In a study on rheumatoid arthritis by Michael Croft and colleagues, interactions between TNFSF ligands and TNFRSF receptors were observed among APCs, B cells, and T cells of the immune system (247).

Upon antigen stimulation, T cells receive signals through TNFRSF members such as OX40, GITR, DR3, CD27, and 4-1BB, which promote follicular helper T (TFH) cell differentiation, regulating antibody responses and cytokine expression linked to histopathology. APCs, DCs and macrophages, enhance T cell responses by upregulating MHC molecules, co-stimulatory ligands, and inflammatory cytokines via CD40 signaling. Additionally, reverse signaling through membrane-bound TNFSF ligands on DCs, macrophages, and B cells enhances inflammatory cytokine production and supports B cell differentiation (247).

### OX40

OX40, also known as TNFRSF4 (tumor necrosis factor receptor superfamily, member 4), is predominantly expressed on the surface of activated CD4+ and CD8+ T cells. Binding of OX40 to its ligand, OX40L, stimulates the activation of CD8+ T cells and enhances various T cell functions, including cytokine production, proliferation, and survival. OX40 antibody activators (agonists) have been shown to reduce intratumoral Tregs and improve anti-tumor activity. Structurally, OX40 is a type 1 transmembrane glycoprotein, primarily expressed by tregs and, upon activation, also expressed by effector T cells (248).

OX40L, the ligand for OX40, was initially identified on HTLV-1-transformed T cells and is also known as pg34. It is predominantly expressed on APCs but can also be found on NK cells, mast cells, and activated T cells. The interaction between OX40 and OX40L facilitates the migration of activated T cells into tissues in response to inflammatory signals.

The OX40/OX40L interaction recruits TNFR-associated factors (TRAFs) within the intracellular region of OX40, forming a signaling complex that includes IKKα, IKKβ, PI3K, and PKB (Akt) (249). OX40 synergizes with TCR signaling, enhancing NFAT entry into the nucleus by increasing intracellular Ca2+ levels (250). OX40 signaling activates both the classical NF-κB1 pathway and the non-classical PI3K/PKB, NFAT pathway, and NF-κB2 pathway (251). This regulation controls genes involved in T-cell division and survival, promotes cytokine gene transcription, and increases cytokine receptor expression, which is crucial for cell survival (252). Additionally, OX40 signaling leads to the downregulation of CTLA-4 and Foxp3 and induces the expression of anti-apoptotic proteins (Bcl-2, Bcl-xL, and Bfl-1) and cell cycle progression proteins (Survivin) (253).IL-33, released by barrier-disrupted epidermal keratinocytes, stimulates type 2 innate lymphoid cells (ILC2s) and DCs to express OX40L. Moreover, the OX40-OX40L signaling pathway also plays a role in regulating IL-22 production in T cells (197).

Studies have analyzed tumor tissues from mouse models of B-cell lymphomas and human cases of condylomatous and follicular lymphomas, revealing high expression of OX40 and CTLA-4 on the surface of tumor-specific Tregs (CD4+, Foxp3+) (254). OX40 has emerged as a specific biomarker in various cancers. For example, high expression of OX40 in primary ovarian immune cells and recurrent tumor cells is associated with increased chemotherapy sensitivity, while patients lacking OX40 expression are more prone to relapse (255). In patients with cutaneous melanoma, OX40 expression in T cells from sentinel lymph nodes negatively correlates with poor prognostic features such as tumor size, ulceration, and lymph node involvement (256).

Given its role in enhancing the immune response to tumors, several therapeutic strategies have been developed to stimulate the OX40 signaling pathway. These include OX40-specific agonistic antibodies, OX40L-Fc fusion proteins, transfection of DCs with OX40L mRNA, and the use of surface-engineered OX40L-expressing tumor cells (257).

### 4-1BB

4-1BB (CD137) is a co-stimulatory immune checkpoint molecule belonging to the TNF receptor superfamily (TNFRSF) and plays a crucial role in regulating the immune response. The CD137 gene, located on chromosome 1p36, is situated near other co-stimulatory TNFRSF members (258). Identified in 1989, 4-1BB is expressed on antigen-activated T cells but not on resting T cells (259). It is also found on DCs, NKs, activated CD4+ and CD8+ T lymphocytes, eosinophils, natural killer T-cells (NKTs), and mast cells (260) though myeloid-derived suppressor cells (MDSCs) do not express this molecule. Additionally, 4-1BB is present on various tumor cells, including human leukemia cells and several lung tumor cell lines. Its ligand, 4-1BBL, is expressed on some APCs such as B lymphocytes, macrophages, DCs, and activated T cells (261). Anti-4-1BB antibodies have shown the ability to activate cytotoxic T cells and enhance γ-interferon (IFN-γ) production. Both dual and multi-specific antibodies targeting 4-1BB are demonstrating significant potential in cancer therapy (40).

4-1BB recruits TNFR-associated factors TRAF1 and TRAF2, forming a heterotrimeric complex that activates the c-Jun N-terminal kinase (JNK) and extracellular signal-regulated kinase (ERK) pathways, while also enhancing signaling through the β-catenin and AKT pathways. Additionally, 4-1BB signaling is regulated by the master transcription factor NF-κB, which promotes cytokine production and secretion. NF-κB activation further enhances CD8+ T lymphocyte survival by upregulating the expression of anti-apoptotic genes Bcl-xL and Bfl-1 (262).

Dual and multi-specific antibodies targeting 4-1BB have shown significant potential in cancer therapy. The human-derived 4-1BB is a type I transmembrane receptor characterized by four extracellular cysteine-rich domains, a short transmembrane domain, and a C-terminal cytoplasmic domain essential for binding adaptor proteins and facilitating signaling. Its ligand, 4-1BBL, is a type II transmembrane protein presented in a soluble form. It consists of a short N-terminal cytoplasmic region, a transmembrane domain, and an extracellular domain that binds 4-1BB (263). The 4-1BB monomer is elongated, with four cysteine-rich domains arranged linearly. Binding of 4-1BBL to 4-1BB induces signaling through TRAF1 and TRAF2, activating the NF-κB, AKT, p38 MAPK, and ERK pathways (264).

CD137 and/or CD137L agonists stimulate the production of several inflammatory cytokines, such as IL-6, TNF-α, and MCP-1, in adipocytes and macrophages (265). Cross-linking CD137 on B cells enhances immune signaling and induces B cell proliferation (266).

Depletion of DCs *in vivo* significantly diminishes the level of cytotoxic T lymphocyte (CTL) stimulation, thereby impairing the overall efficacy of 4-1BB antibodies. These antibodies activate various immune cells through 4-1BB signaling, modulating T cell activity, inducing cytokine production, and preventing activation-induced cell death (AICD), ultimately enhancing CTL activity. 4-1BB is considered a highly promising target in immuno-oncology and remains one of the most attractive T-cell co-stimulatory receptors within the TNF receptor superfamily (TNFRSF). Phase I trials for next-generation 4-1BB targeting agents are currently focusing on mitigating hepatotoxicity while maintaining therapeutic efficacy (267).

## Outstanding questions and concluding remarks

In summary, the co-inhibitory and co-stimulatory pathways of immune checkpoint proteins are crucial for maintaining immune homeostasis, preventing infections, and avoiding autoimmunity. These pathways regulate not only the activation of naïve T cells but also the immune responses of memory cells and Tregs. Although significant progress has been made in understanding the immunoregulatory roles of these pathways, challenges remain, such as adverse effects associated with immune checkpoint inhibition during antibody drug development, including hepatotoxicity observed with 4-1BB agonists (198).

Currently, combination therapies targeting immune checkpoints have been widely adopted for treating various diseases. Additionally, it has been observed that immune checkpoint expression can be modulated by the tumor microenvironment—for instance, pH levels influence VISTA expression (99). While previous research has largely concentrated on T-cell responses, emerging data on TIM-1’s stimulatory effects on B cells offer new biological insights and strategies (210). This evolving knowledge enhances our understanding of the efficacy of current immunotherapies and opens avenues for developing novel therapeutic approaches. The FDA’s approval of CTLA-4, PD-1, and PD-L1 antibodies underscores the therapeutic potential of a deeper understanding of co-inhibitory pathways, with agonistic antibodies for autoimmune diseases showing promise. Continued research will refine our grasp of these pathways in health and disease, leading to more effective and safe treatments for various immune-mediated conditions.

Immune checkpoint combination therapy represents a pivotal advancement in tumor immunotherapy, offering significant clinical potential. By simultaneously targeting multiple immune checkpoints, such as PD-1/PD-L1 and CTLA-4, this approach overcomes the limitations of single-target therapies and amplifies anti-tumor immune responses. For instance, PD-1/PD-L1 inhibitors restore effector TCR functionality, while CTLA-4 inhibitors promote the activation of naïve T cells (46, 47). The synergistic effects of these pathways have demonstrated substantial improvements in therapeutic efficacy. Combination therapies have achieved high ORRs and durable efficacy in solid tumors, such as melanoma and NSCLC, leading to significant improvements in long-term OS (88, 89).

Furthermore, combining emerging immune checkpoint molecules, such as LAG-3, TIGIT, and TIM-3, with classical checkpoint inhibitors has opened new avenues for immunotherapy. For example, the combination of LAG-3 and PD-1 inhibition has shown notable efficacy across various tumor models (225, 226). Similarly, strategies targeting TIGIT in combination with PD-L1 inhibitors have demonstrated promising potential in both solid tumors and hematologic malignancies (227, 228).

The primary advantage of immune checkpoint combination therapies lies in their ability to enhance therapeutic efficacy through multi-targeted interventions while addressing the resistance often encountered in monotherapies. However, this approach also presents challenges, including increased toxicity and the complexity of designing individualized treatment regimens for patients. Future research will prioritize optimizing combination strategies, selecting precise checkpoint combinations, and integrating biomarkers to predict treatment responses and patient outcomes.

In conclusion, immune checkpoint combination therapy is a transformative innovation in tumor immunotherapy. It not only provides novel therapeutic options for various malignancies but also lays a solid foundation for the development of precision medicine. This approach highlights its vast potential in advancing anti-tumor therapy and improving patient outcomes.

Checkpoint-blocking immunotherapies have demonstrated efficacy across a broad range of cancers and have significantly impacted clinical practice in oncology. Among the next-generation immune checkpoint targets—such as LAG-3, the Ig domain-containing VISTA, TIM-3, TIGIT, B7-H3, and SIRPα—each shows promising therapeutic potential, though it remains uncertain which will become the next major breakthrough like PD-1.
